# Retrobulbar Hemodynamic Alterations in Diabetes Mellitus: Doppler Evaluation of the Ophthalmic and Central Retinal Arteries

**DOI:** 10.7759/cureus.111625

**Published:** 2026-06-27

**Authors:** Kotresh N S, Vinutha H, ApoorvaPooja K, Prajwal M P

**Affiliations:** 1 Radiology, Sri Siddhartha Medical College, Tumkur, IND

**Keywords:** central retinal artery, diabetes mellitus, diabetic retinopathy, doppler ultrasound, ophthalmic artery, retrobulbar circulation

## Abstract

Background: Diabetes mellitus causes microvascular alterations that contribute to the development of diabetic retinopathy. Hemodynamic changes in retrobulbar circulation may occur before clinically detectable retinal damage.

Purpose: This study aimed to compare Doppler hemodynamic indices of the ophthalmic artery (OA) and central retinal artery (CRA) between diabetic participants and non-diabetic controls.

Materials and methods: This cross-sectional observational study included 76 participants (38 diabetic participants and 38 non-diabetic controls), comprising 152 eyes analyzed at the eye level. Color Doppler imaging was used to measure peak systolic velocity (PSV), end-diastolic velocity (EDV), resistive index (RI), and systolic/diastolic (S/D) ratio in the OA and CRA. Group comparisons were performed using an independent samples t-test, with statistical significance set at p < 0.05.

Results: Diabetic participants demonstrated significantly higher vascular resistance in retrobulbar arteries. OA-RI was significantly higher in diabetic participants (0.80 ± 0.03) than in non-diabetic controls (0.71 ± 0.01; p < 0.001). CRA-RI was similarly elevated (0.70 ± 0.04 vs. 0.62 ± 0.01; p < 0.001). Retrobulbar blood flow velocities were significantly reduced in diabetic participants, including OA-PSV (19.38 ± 2.71 vs. 29.18 ± 1.42 cm/s; p < 0.001) and OA-EDV (3.84 ± 1.04 vs. 8.49 ± 0.53 cm/s; p < 0.001). CRA-PSV (9.02 ± 1.23 vs. 13.28 ± 0.64 cm/s; p < 0.001) and CRA-EDV (2.87 ± 0.82 vs. 5.05 ± 0.33 cm/s; p < 0.001) were also significantly reduced. S/D ratios were significantly elevated in both vessels among diabetic participants.

Conclusions: Diabetes is associated with increased vascular resistance and reduced blood flow velocities in the OA and CRA. These findings suggest that color Doppler imaging may help identify early hemodynamic alterations in the ocular circulation associated with diabetic retinal disease.

## Introduction

Diabetes mellitus is a major global health problem and a leading cause of microvascular complications affecting multiple organ systems. The global prevalence of diabetes has increased substantially over recent decades, reflecting changes in lifestyle, aging populations, and increasing urbanization [1]. Among the various complications of diabetes, diabetic retinopathy (DR) remains one of the most important causes of visual impairment and preventable blindness worldwide [2]. Epidemiological studies have shown that the prevalence of DR among diabetic participants is considerable, with the risk increasing with longer duration of disease and poor glycemic control [3]. Large population-based studies such as the Wisconsin Epidemiologic Study of Diabetic Retinopathy have highlighted the substantial burden of retinal complications associated with diabetes [4].

The pathogenesis of DR involves complex microvascular alterations resulting from chronic hyperglycemia. Structural and functional changes in retinal microvasculature include endothelial dysfunction, basement membrane thickening, capillary non-perfusion, and pericyte loss, ultimately leading to impaired retinal perfusion and ischemia [5]. These microvascular abnormalities not only affect the retinal circulation but may also influence hemodynamics in the orbital vessels supplying the eye.

Color Doppler imaging has emerged as a valuable non-invasive technique for evaluating ocular and retrobulbar blood flow. By measuring parameters such as peak systolic velocity (PSV), end-diastolic velocity (EDV), and resistive index (RI), color Doppler imaging enables assessment of vascular resistance and perfusion in the orbital arteries, including the ophthalmic artery (OA) and central retinal artery (CRA) [6]. Early studies demonstrated that Doppler ultrasound could detect alterations in orbital blood flow in patients with DR, suggesting that vascular changes may occur before clinically detectable retinal damage [7].

Several investigators have subsequently evaluated retrobulbar hemodynamics in diabetic participants using color Doppler imaging. These studies have reported reduced blood flow velocities and increased vascular resistance in orbital vessels among patients with DR compared with healthy individuals [8,9]. Furthermore, Doppler studies have demonstrated correlations between retinal microvascular perfusion and circulatory parameters of retrobulbar vessels, supporting the concept that orbital hemodynamics may reflect underlying retinal vascular pathology [10].

Assessment of ocular blood flow parameters also requires reliable reference values and standardized Doppler measurement techniques. Previous investigations have established normal ranges and variability of Doppler parameters in orbital vessels, which provide an important basis for interpreting hemodynamic changes in pathological conditions [11].

Given the growing burden of diabetes and the importance of early detection of vascular alterations associated with DR, evaluating orbital hemodynamics with color Doppler imaging may provide useful insights into the pathophysiology of ocular vascular changes in diabetic participants. Although previous studies have demonstrated altered retrobulbar hemodynamics in diabetic participants, further evaluation of Doppler findings in relation to DR status and clinical indicators such as HbA1c and duration of diabetes may provide additional clinically relevant information. Therefore, the present study aimed to assess Doppler indices of the OA and CRA in diabetic participants and non-diabetic controls and to analyze hemodynamic alteration patterns associated with DR.

## Materials and methods

Study design and participants

This cross-sectional observational study was conducted in the Department of Radiodiagnosis at a tertiary care teaching hospital between January 2025 and February 2026. The Institutional Ethics Committee of Sri Siddhartha Medical College issued approval SSMC/MED/IEC-87, and written informed consent was obtained from all participants in accordance with the principles of the Declaration of Helsinki.

A total of 76 participants were enrolled, comprising 38 diabetic participants with previously diagnosed type 2 diabetes mellitus and 38 non-diabetic controls. Both eyes of each participant were examined, resulting in a total of 152 eyes included for analysis. Diabetic status was confirmed based on medical history, clinical records, and laboratory investigations, including fasting blood glucose levels. The control group consisted of individuals without a history of diabetes and with normal fasting blood glucose levels.

Participants in the diabetic group were between 35 and 64 years of age and had a confirmed diagnosis of type 2 diabetes mellitus. The control group was age- and sex-matched as closely as possible to the diabetic group. Individuals with ocular conditions known to influence ocular blood flow, such as glaucoma, uveitis, high myopia (>6 diopters), or previous ocular surgery, were excluded. Patients with systemic conditions that could affect vascular hemodynamics, including uncontrolled hypertension, cardiovascular disease, or cerebrovascular disease, were also excluded. Pregnant patients, individuals with media opacity that prevents adequate ocular evaluation, and those unwilling to provide consent were excluded from the study.

All participants underwent clinical evaluation, including demographic data collection, blood pressure measurement, and fasting blood glucose estimation. In diabetic participants, HbA1c levels and diabetes duration were recorded. A comprehensive ophthalmic examination was performed in all participants, including visual acuity assessment, slit-lamp examination, intraocular pressure measurement, and dilated fundus examination. In diabetic participants, DR was graded using standard clinical criteria based on fundoscopic findings.

Doppler ultrasonography

Color Doppler imaging of the orbital vessels was performed using a high-resolution ultrasound system equipped with a linear array transducer operating at 7-14 MHz. All examinations were performed by a single experienced radiologist to minimize interobserver variability. Participants were examined in the supine position with their eyelids closed, and acoustic gel was applied over the eyelid to ensure adequate acoustic coupling. The transducer was placed gently on the eyelid with minimal pressure to avoid alteration of ocular blood flow.

The mechanical index was maintained at ≤0.6 to ensure ocular safety. Color Doppler gain and wall filter settings were optimized for the detection of low-velocity orbital blood flow. Doppler angle correction was applied and maintained below 20° to improve the accuracy of velocity measurements.

The OA was identified medial to the optic nerve, approximately 10-15 mm posterior to the globe, while the CRA was visualized within the optic nerve shadow. Spectral Doppler waveforms were obtained from each vessel using a small sample volume (approximately 1-2 mm). Doppler parameters recorded included PSV, EDV, RI, and systolic-to-diastolic (S/D) ratio. The RI was automatically calculated by the ultrasound system using the standard formula RI = (PSV − EDV) / PSV.

For each vessel, three consecutive spectral waveforms were obtained, and the mean value was used for analysis. A Doppler examination was performed according to previously described methods for evaluating orbital blood flow [11].

Statistical analysis

Statistical analysis was performed using SPSS Statistics version 24.0 (IBM Corp. Released 2016. IBM SPSS Statistics for Windows, Version 24.0. Armonk, NY: IBM Corp.). Continuous variables were expressed as mean ± standard deviation (SD), while categorical variables were presented as frequencies and percentages. Because Doppler measurements were obtained from both eyes of each participant, analyses were performed at the eye level as commonly reported in ophthalmic Doppler studies evaluating retrobulbar hemodynamics. Normality of continuous variables was assessed using the Kolmogorov-Smirnov test. As the variables were normally distributed, comparisons between diabetic participants and non-diabetic controls were performed using the independent-samples t-test. Categorical variables were compared using the chi-square test. Pearson’s correlation coefficient was used to evaluate relationships between Doppler parameters and clinical variables such as duration of diabetes and HbA1c levels. A p-value < 0.05 was considered statistically significant. All statistical tests were two-tailed.

## Results

A total of 76 participants were included in the study, comprising 38 diabetic participants with type 2 diabetes mellitus and 38 non-diabetic controls. Both eyes of each participant were evaluated, resulting in a total of 152 eyes analyzed (76 eyes in the diabetic group and 76 eyes in the control group). The demographic and clinical characteristics of the study population are summarized in Table 1.

**Table 1:** Demographic and clinical characteristics of study participants Values are expressed as mean ± SD unless otherwise indicated. Group comparisons were performed using the independent-samples t-test for continuous variables and the chi-square test for categorical variables. p < 0.05 indicates statistical significance. SD: standard deviation, HbA1c: glycated hemoglobin

Parameter	Non-diabetic (n = 38)	Diabetic (n = 38)	p-value
Age (years), mean ± SD	47.6 ± 7.2	52.8 ± 6.8	0.067
Gender	-	-	0.821
Male, n (%)	18 (47.4%)	19 (50.0%)	-
Female, n (%)	20 (52.6%)	19 (50.0%)	-
Body mass index (kg/m²), mean ± SD	23.8 ± 2.9	24.9 ± 3.1	0.112
Fasting blood glucose (mg/dL), mean ± SD	92.4 ± 8.6	168.7 ± 42.3	<0.001*
Systolic blood pressure (mmHg), mean ± SD	121.4 ± 8.2	124.6 ± 10.1	0.138
Diastolic blood pressure (mmHg), mean ± SD	78.2 ± 5.6	79.4 ± 6.8	0.402
Duration of diabetes (years), mean ± SD	-	8.4 ± 4.2	-
HbA1c (%), mean ± SD	-	8.2 ± 1.6	-

The mean age of participants in the diabetic group was 52.8 ± 6.8 years, while the control group had a mean age of 47.6 ± 7.2 years. The overall mean age of the study population was 50.2 ± 7.3 years (range 35-64 years). The difference in age between the two groups was not statistically significant (p = 0.067). Gender distribution was comparable between groups, with 19 males and 19 females in the diabetic group and 18 males and 20 females in the control group (p = 0.821). Mean body mass index was 24.9 ± 3.1 kg/m² in diabetic participants and 23.8 ± 2.9 kg/m² in non-diabetic controls (p = 0.112). Fasting blood glucose levels were significantly higher in the diabetic group (168.7 ± 42.3 mg/dL) compared with the control group (92.4 ± 8.6 mg/dL; p < 0.001). The mean duration of diabetes among diabetic participants was 8.4 ± 4.2 years, and the mean HbA1c level was 8.2 ± 1.6%.

**Figure 1:**
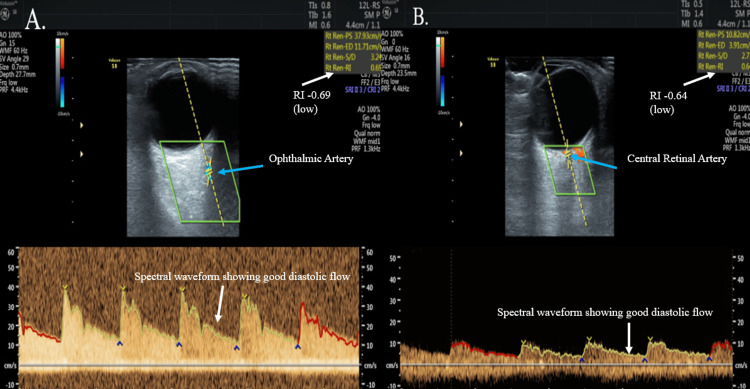
Normal retrobulbar Doppler waveforms in a non-diabetic individual. (A) Spectral Doppler waveform of the OA (blue arrow) demonstrating normal low-resistance flow with a sharp systolic upstroke and continuous diastolic flow (white arrow). (B) Spectral Doppler waveform of the CRA (blue arrow) obtained within the optic nerve shadow showing preserved diastolic flow and normal RI (white arrow). These waveforms illustrate normal retrobulbar hemodynamics in the OA and CRA OA: ophthalmic artery, CRA: central retinal artery, RI: resistive index

Doppler evaluation of the OA demonstrated significant differences in hemodynamic parameters between diabetic participants and non-diabetic controls (Table 2). PSV in the OA was significantly lower in the diabetic group (19.38 ± 2.71 cm/s, n = 76 eyes) compared with the control group (29.18 ± 1.42 cm/s, n = 76 eyes; p < 0.001). Similarly, EDV was significantly reduced in diabetic participants (3.84 ± 1.04 cm/s) compared with non-diabetic controls (8.49 ± 0.53 cm/s; p < 0.001). In contrast, the RI of the OA was significantly higher in the diabetic group (0.80 ± 0.03) than in the control group (0.71 ± 0.01), indicating increased downstream vascular resistance (p < 0.001). The systolic-to-diastolic ratio was also significantly elevated in the diabetic group (5.15 ± 0.68) compared with the control group (3.44 ± 0.05; p < 0.001). Representative spectral Doppler waveforms of the OA and CRA in a non-diabetic control are shown in Figure 1.

**Table 2:** Color Doppler parameters of the OA Values are expressed as mean ± SD. Comparisons between groups were performed using the independent samples t-test. p < 0.05 indicates statistical significance. SD: standard deviation, OA: ophthalmic artery, PSV: peak systolic velocity, EDV: end-diastolic velocity, RI: resistive index, S/D: systolic/diastolic ratio

Parameter	Non-diabetic (n = 76 eyes)	Diabetic (n = 76 eyes)	p-value
PSV (cm/s)	29.18 ± 1.42	19.38 ± 2.71	<0.001*
EDV (cm/s)	8.49 ± 0.53	3.84 ± 1.04	<0.001*
RI	0.71 ± 0.01	0.80 ± 0.03	<0.001*
S/D	3.44 ± 0.05	5.15 ± 0.68	<0.001*

**Figure 2:**
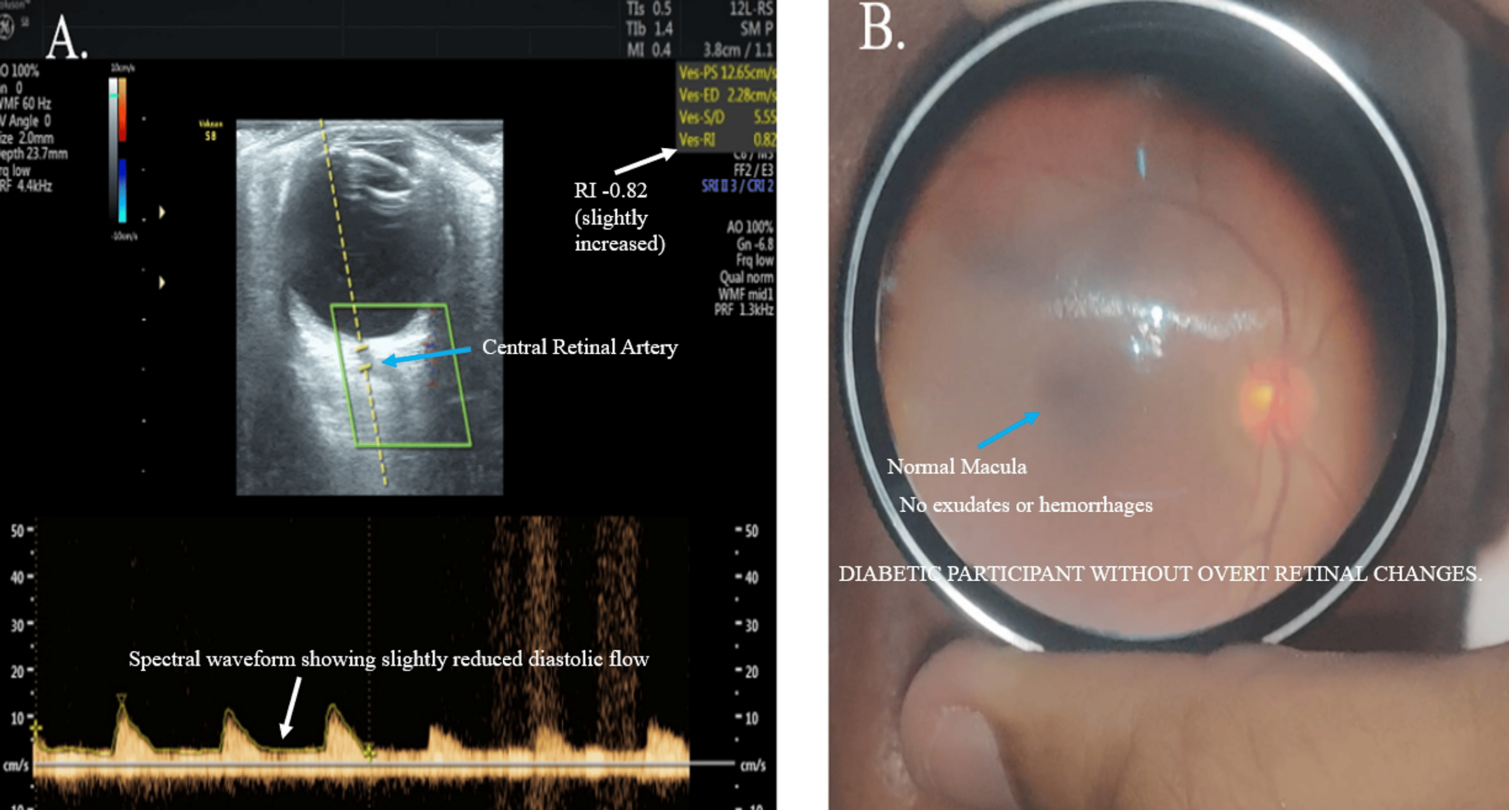
Altered retrobulbar hemodynamics in a patient with diabetes mellitus without overt retinopathy. (A) Spectral Doppler waveform of the CRA (blue arrow) showing decreased EDV and elevated RI (white arrow). (B) Fundus photograph of the same eye demonstrating absence of clinically detectable DR (normal macula (blue arrow), no exudates or hemorrhages). These findings correspond to the reduced retrobulbar blood flow velocities and increased vascular resistance observed in diabetic participants without overt retinal changes CRA: central retinal artery, EDV: end-diastolic velocity, RI: resistive index, DR: diabetic retinopathy

Similar trends were observed in the Doppler parameters of the CRA (Table 3). PSV in the CRA was significantly lower in diabetic participants (9.02 ± 1.23 cm/s, n = 76 eyes) compared with non-diabetic controls (13.28 ± 0.64 cm/s, n = 76 eyes), with a mean difference of 4.26 cm/s (p < 0.001). EDV was also significantly reduced in diabetic participants (2.87 ± 0.82 cm/s) compared with non-diabetic controls (5.05 ± 0.33 cm/s; p < 0.001). The RI of the CRA was significantly higher in diabetic participants (0.70 ± 0.04) than in non-diabetic controls (0.62 ± 0.01; p < 0.001). Likewise, the systolic-to-diastolic ratio was significantly elevated in the diabetic group (3.34 ± 0.44) relative to the control group (2.63 ± 0.05; p < 0.001). Representative Doppler waveforms of the CRA in a diabetic participant without overt retinopathy, along with the corresponding fundus photograph, are illustrated in Figure 2.

**Table 3:** Color Doppler parameters of the CRA Values are expressed as mean ± SD. Comparisons between groups were performed using the independent samples t-test. p < 0.05 indicates statistical significance. SD: standard deviation, CRA: central retinal artery, PSV: peak systolic velocity, EDV: end-diastolic velocity, RI: resistive index, S/D: systolic/diastolic ratio

Parameter	Non-diabetic (n = 76 eyes)	Diabetic (n = 76 eyes)	p-value
PSV (cm/s)	13.28 ± 0.64	9.02 ± 1.23	<0.001*
EDV (cm/s)	5.05 ± 0.33	2.87 ± 0.82	<0.001*
RI	0.62 ± 0.01	0.70 ± 0.04	<0.001*
S/D	2.63 ± 0.05	3.34 ± 0.44	<0.001*

Among diabetic participants (76 eyes), DR was identified in 44 eyes, while 32 eyes showed no evidence of retinopathy on fundus examination. Comparisons between eyes with and without DR were performed using an independent-samples t-test. Doppler parameters showed more pronounced hemodynamic alterations in eyes with DR than in those without.

**Figure 3:**
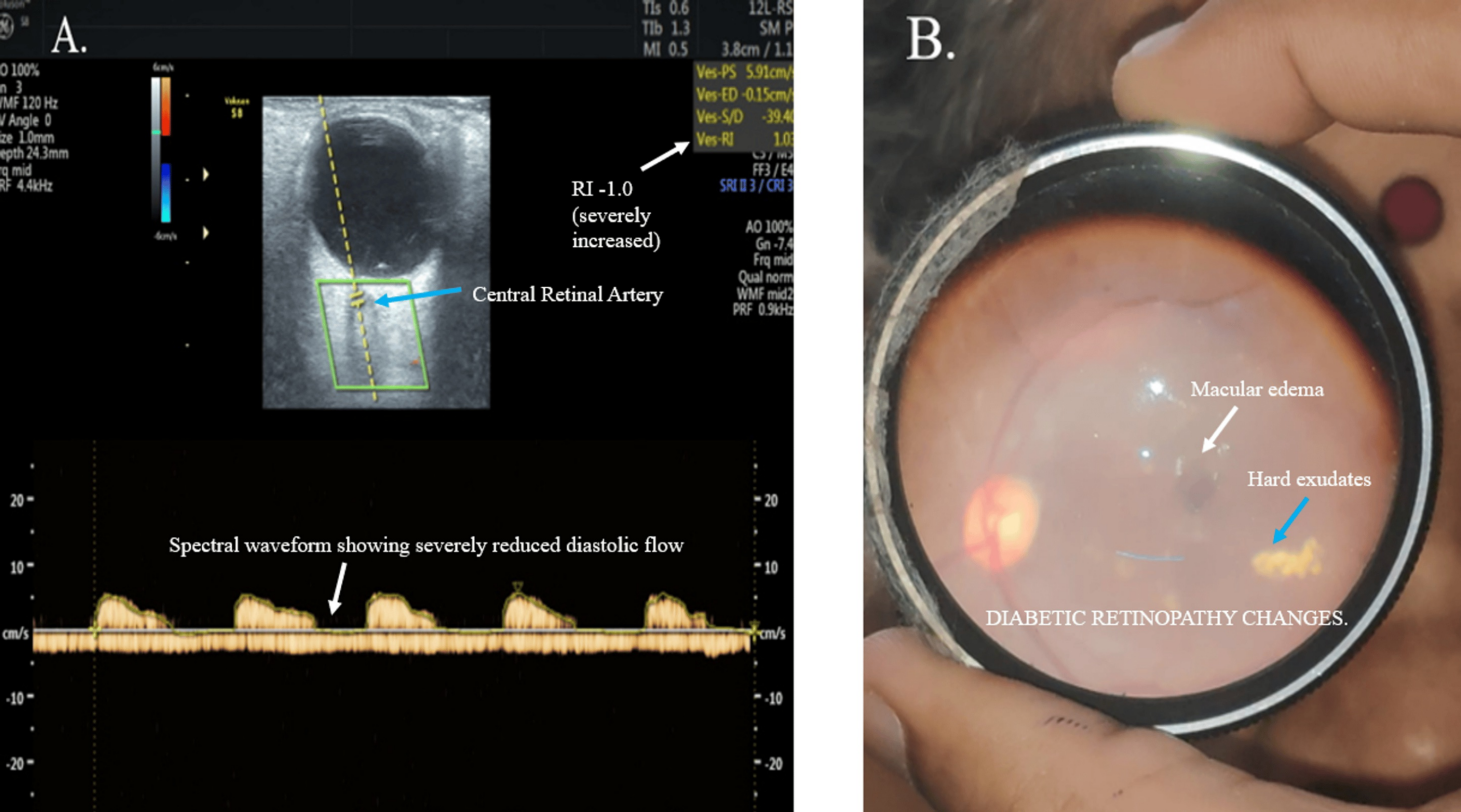
Representative retrobulbar Doppler waveforms in DR. (A) Spectral Doppler waveform of the CRA (blue arrow) showing reduced EDV and elevated vascular resistance (white arrow). (B) Fundus photograph of the same eye demonstrating severe non-proliferative DR with hard exudates and clinically significant macular edema (white arrow). These images illustrate the more pronounced retrobulbar hemodynamic alterations observed in eyes with DR CRA: central retinal artery, DR: diabetic retinopathy, EDV: end-diastolic velocity

In eyes with retinopathy (n = 44), OA PSV was significantly lower (17.89 ± 2.45 cm/s) than in eyes without retinopathy (21.42 ± 2.18 cm/s; n = 32, p < 0.001). EDV was also reduced in the retinopathy group (3.24 ± 0.92 cm/s vs 4.68 ± 0.89 cm/s; p < 0.001). Correspondingly, the RI was higher in eyes with retinopathy (0.82 ± 0.03) than in those without retinopathy (0.78 ± 0.02; p < 0.001). Representative Doppler waveforms of the CRA in eyes with DR, along with the corresponding fundus photograph, are shown in Figure 3.

Correlation analysis demonstrated a moderate positive correlation between the duration of diabetes and the OA RI (r = 0.412, p = 0.001) and the CRA RI (r = 0.384, p = 0.003). HbA1c levels showed a negative correlation with CRA EDV (r = −0.356, p = 0.008) and a positive correlation with OA RI (r = 0.298, p = 0.032). Age demonstrated weak correlations with Doppler parameters that did not reach statistical significance.

## Discussion

The present study demonstrates significant alterations in retrobulbar hemodynamics in patients with diabetes mellitus compared with non-diabetic controls. Doppler ultrasound evaluation of the OA and CRA revealed reduced PSV and EDV and increased RI values in diabetic participants. These findings support the concept that diabetes-related microvascular dysfunction extends beyond the retinal capillary bed and affects the orbital circulation supplying the eye.

DR is a well-recognized microvascular complication of chronic hyperglycemia and remains a major cause of visual impairment worldwide [2,3]. Persistent metabolic injury results in endothelial dysfunction, basement membrane thickening, capillary dropout, and pericyte loss, ultimately leading to impaired retinal perfusion and ischemia [5]. Because the OA and CRA represent the principal arterial supply to ocular structures, alterations in their hemodynamic characteristics may reflect early vascular compromise associated with diabetic microangiopathy.

More recent clinical studies demonstrated that orbital circulation may be altered in diabetic participants and that color Doppler imaging can detect functional hemodynamic abnormalities in retrobulbar vessels [6-10]. These studies suggested that changes in retrobulbar blood flow may accompany or even precede clinically detectable retinal microvascular damage.

In the present study, diabetic participants demonstrated reduced blood flow velocities and increased vascular resistance in both the OA and CRA. Similar findings have been reported by Kraśnicki et al., who observed reduced retrobulbar blood flow velocities in diabetic participants compared with non-diabetic controls using color Doppler imaging [12]. These authors suggested that diabetes-related vascular dysfunction may affect orbital circulation even before advanced retinal pathology becomes clinically apparent.

More recent clinical studies have further confirmed these observations. Divya et al. reported significantly reduced PSV and EDV, along with increased RI values, in the retrobulbar vessels of patients with type 2 diabetes mellitus compared with healthy individuals [13]. Similarly, Madhpuriya et al. demonstrated decreased retrobulbar blood flow velocities and increased vascular resistance in diabetic participants using color Doppler imaging [14]. Our findings are consistent with these reports, demonstrating significantly reduced blood flow velocities and increased resistive indices in both the OA and CRA. Furthermore, diabetic eyes with retinopathy in the present study exhibited more pronounced hemodynamic alterations than eyes without retinopathy, supporting the association between worsening retinal disease and increasing vascular resistance.

Evidence from pooled analyses also supports these observations. A meta-analysis by Meng et al. evaluating retrobulbar blood flow parameters in diabetic participants confirmed significantly reduced PSV and EDV, along with elevated RI values, compared with non-diabetic controls [15]. These findings indicate that alterations in orbital hemodynamics represent a reproducible feature of diabetic ocular disease across multiple independent studies.

More recently, Noureldine et al. demonstrated measurable alterations in orbital blood flow parameters in diabetic participants with and without DR, suggesting that retrobulbar vascular abnormalities may occur even before advanced retinal damage becomes clinically evident [16]. Our observations are consistent with these findings, as diabetic participants without overt retinopathy also exhibited abnormal Doppler parameters. In contrast, eyes with DR showed significantly greater reductions in blood flow velocities and higher vascular resistance. These findings further support the progressive nature of ocular vascular dysfunction in diabetes mellitus.

Several additional Doppler investigations have reported abnormalities in retrobulbar circulation in diabetic participants, including reduced blood flow velocities and increased vascular resistance in orbital vessels [17-20]. Collectively, these investigations provide strong evidence that diabetes-related vascular dysfunction affects both the retinal microcirculation and the larger retrobulbar arteries that supply the eye.

An important observation in the present study was the more pronounced hemodynamic alterations in eyes with DR than in those without retinopathy. Eyes with retinopathy demonstrated lower EDV and higher RI values in the OA compared with eyes without retinopathy. These findings suggest that progressive microvascular damage associated with DR may increase vascular resistance in the ocular circulation, potentially contributing to retinal hypoperfusion and ischemia.

Correlation analysis in the present study demonstrated significant relationships between Doppler parameters and clinical indicators of diabetic disease severity. The duration of diabetes showed a moderate positive correlation with the RI values in both the OA and CRA. Similarly, higher HbA1c levels were associated with increased vascular resistance and reduced EDV in the CRA. These findings support the concept that chronic metabolic injury and poor glycemic control contribute to progressive deterioration of ocular vascular function in diabetic participants.

Although previous studies have reported alterations in retrobulbar blood flow in diabetic participants, the present study contributes to the existing evidence by simultaneously evaluating the OA and CRA using a standardized Doppler protocol. In addition to comparing diabetic participants with non-diabetic controls, diabetic eyes were further evaluated for the presence or absence of DR, and Doppler findings were analyzed in relation to HbA1c levels and duration of diabetes. These observations provide additional clinical context on retrobulbar hemodynamic alterations associated with diabetes and complement the findings of previous Doppler studies.

The clinical implications of these findings are noteworthy. Doppler ultrasound is a non-invasive and widely available imaging modality that allows real-time evaluation of orbital vascular hemodynamics. Measurement of blood flow velocities and resistive indices in retrobulbar vessels may provide useful information on early vascular changes associated with diabetes. Identification of such hemodynamic abnormalities may help identify patients at risk for developing DR or experiencing progression of existing retinal disease. These findings support the potential role of Doppler ultrasound as a non-invasive adjunct imaging technique for evaluating early ocular vascular alterations in patients with diabetes.

Several limitations of the present study should be acknowledged. First, the study was conducted at a single center with a relatively modest sample size, which may limit the generalizability of the findings. Second, Doppler measurements were analyzed at eye level because both eyes of each participant were evaluated, which could introduce inter-eye correlations. Third, although Doppler ultrasound provides information on blood flow velocities and vascular resistance, it does not directly measure retinal microvascular perfusion. Future multicenter prospective studies involving larger patient populations and longer follow-up are required to validate these findings. Integration of color Doppler parameters with established clinical indicators such as HbA1c, duration of diabetes, retinal examination, and multimodal retinal imaging, including optical coherence tomography and optical coherence tomography angiography, may further improve the assessment of ocular vascular changes and help identify patients who may benefit from closer ophthalmic surveillance and optimized systemic disease control.

## Conclusions

Color Doppler ultrasound demonstrated significantly reduced blood flow velocities and increased vascular resistance in both the OA and CRA among diabetic participants compared with non-diabetic controls. These hemodynamic alterations were more pronounced in eyes with DR, supporting the association between retrobulbar vascular dysfunction and the severity of retinal disease. The present findings further support the potential role of color Doppler imaging as a useful non-invasive adjunct for evaluating ocular vascular changes in diabetes, particularly when interpreted alongside routine clinical and ophthalmologic assessment.
